# Non-invasive electrophysiology in glaucoma, structure and function—a review

**DOI:** 10.1038/s41433-021-01603-0

**Published:** 2021-06-11

**Authors:** Khaldoon O. Al-Nosairy, Michael B. Hoffmann, Michael Bach

**Affiliations:** 1 Department of Ophthalmology, Otto-von-Guericke University, Magdeburg, Germany; 2 Center for Behavioral Brain Sciences, Magdeburg, Germany; 3 Faculty of Medicine, Eye Center, Medical Center—University of Freiburg, Freiburg im Breisgau, Germany

**Keywords:** Predictive markers, Physiology

## Abstract

Glaucoma, its early diagnosis, and monitoring of interventions remain an ongoing challenge. We here review developments in functional assessment and its relation to morphology, evaluating recent insights in electrophysiology in glaucoma and highlighting how glaucoma research and diagnostics benefit from combined approaches of OCT and electrophysiological investigations. After concise overviews of OCT and non-invasive electrophysiology in glaucoma, we evaluate commonalities and complementarities of OCT and electrophysiology for our understanding of glaucoma. As a specific topic, the dynamic range (floor effects) of the various techniques is discussed.

## 摘要

青光眼的早期诊断和干预的监测仍然是一个持续性的挑战。在此, 我们回顾了功能评估及其与形态学关系的进展, 评价了青光眼电生理学的最新观点和见解, 并强调了联合使用OCT以及无创电生理在青光眼的研究方面获益。在简要综述了OCT和无创电生理学在青光眼中的应用后, 我们评估了OCT和电生理学在我们理解青光眼方面的共同点和互补性。除此之外, 我们将各种技术的动态范围 (天花板效应) 作为一个特定的主题进行了讨论。

## Introduction

Glaucoma is a globally leading cause of irreversible blindness [1] and is characterized by progressive retinal ganglion cell (RGC) changes associated with visual field (VF) loss that might end up in functional disability. The overarching goal of glaucoma management is the prevention of visual disability through the early detection of glaucomatous damage and monitoring its progression. Several tools are at hand that allows for reproducible assessment of functional visual loss. Static automated perimetry (SAP) is routinely used clinically for this purpose albeit variable and subjective. Important complements are objective measures of vision, i.e., ocular coherence tomography (OCT) and non-invasive electrophysiology. They are widely adopted in glaucoma research to provide reproducible quantitative surrogate markers and insights into the underlying pathophysiology via structural and vascular assessments (OCT) and functional read-outs (electrophysiology). This review evaluates recent insights in electrophysiology in glaucoma and highlights how glaucoma research and diagnostics benefit from combined approaches of OCT and electrophysiological investigations. We address this in three steps: after concise overviews of (i) OCT and (ii) non-invasive electrophysiology in glaucoma, we evaluate (iii) commonalities and complementarities of OCT and electrophysiology to further our understanding of glaucoma.

## OCT in glaucoma

### Assessing retinal structure with OCT

The loss of RGCs and their axons are the hallmarks of neurodegenerative processes in glaucoma [2]. OCT has optimized and revolutionized the structural assessment of glaucoma and is the imaging modality of choice for glaucoma diagnostics [3]. It offers insights into RGC degeneration in glaucoma at two main retinal sites: (i) peripapillary retina and (ii) macula. (i) At the peripapillary retinal area, OCT scans enable the quantitative and objective assessment of the RGC axonal loss via determining the peripapillary retinal nerve fiber layer thickness (pRNFL), an approach that has an established role in glaucoma management [4, 5]. (ii) The macula, on the other hand, has gained interest in the structural assessment of glaucoma, where evidence has accrued to demonstrate its early involvement in the disease process. In fact, the macula encompasses a high density of RGC (50% of total RGCs) which underscores its importance in glaucoma assessment [5, 6]. Changes of the RGC at the macula and its surrogate structural measure containing RGC bodies and dendrites, i.e., ganglion cell inner plexiform layer (GCIPL), and axons, i.e., the ganglion cell complex (GCC), have been extensively investigated and its diagnostic role is established in glaucoma detection and progression—see reviews [2–4, 7].

### Peripapillary vs macular OCT parameters for glaucoma detection

Diagnostic performance of pRNFL and macular GCIPL and GCC was ascertained in glaucoma, where most of the studies reported comparable area under the curve (AUC) of pRNFL vs GCIPL/GCC measures, i.e., the discriminative performance between glaucoma and controls. In a recent meta-analysis of 150 studies [8], the pooled AUC for early glaucoma detection, i.e., preperimetric glaucoma, was comparable between these measures, i.e., 0.83, 0.76, and 0.80, respectively. In moderate to severe glaucoma, the performance of peripapillary and macular measures was also comparable, i.e., AUC of 0.96 and 0.94, respectively.

### Peripapillary and macular OCT parameters for progression monitoring

Both peripapillary and inner macular thickness might also have a critical role in the timely identification of glaucoma progression to implement therapy and avert permanent visual loss. However, many studies demonstrated the beneficial role of either GCC or GCIPL over pRNFL in monitoring glaucoma—see reviews [3, 9]. At preperimetric and suspect glaucomatous stages, it was reported that patients with abnormal or borderline baseline GCC focal loss volume had a fourfold higher risk for glaucoma conversion over 6 years period [10]. Another study [11] followed patients with established glaucoma and demonstrated a threefold risk of VF progression over 3 years with the presence of abnormal GCC at baseline. In spite of advances in OCT imaging, various factors influence the accurate assessment of the thickness of the various retinal layers, possibly involving changes of glial cells, astrocytes, and Müller cells. This stimulates further explorations next to OCT such as the assessment of vascular and functional retinal measures.

### Assessing retinal vasculature with OCT-A

Optical coherence tomography angiography (OCT-A) is a nascent imaging modality assessing non-invasively the microvasculature of the macula, the peripapillary area, and the optic nerve head that might improve glaucoma management, as reviewed in [12–14]. It is well established that vessel densities (VD), i.e., OCT-A vascular measures, are reduced at these sites in glaucoma [13, 14], where specific OCT-A quantification readouts are currently being established [15]. OCT-A, besides its potential for glaucoma diagnosis, might provide valuable insights about glaucoma pathomechanisms [16–19]. In conclusion, OCT-A offers an objective measure for detection, progression monitoring, and risk assessment in glaucoma [13, 14].

### Combined OCT/OCT-A for disease detection, follow-up, and mechanisms

At the peripapillary area, most of the studies, see reviews [12, 20], found the OCT-A VD and OCT pRNFL to have an AUC for perimetric glaucoma detection above 0.85, while several studies reported comparable AUC, i.e., 0.95, for the macular inner thickness including GCIPL and whole-image (6 × 6 mm²) macular VD. However, the highest discriminatory AUC was obtained for the combined OCT/OCT-A measures. For early glaucoma detection, OCT-A measures showed promising results, which are at least equal to OCT measures. For advanced glaucoma, peripapillary VD demonstrated a less pronounced floor effect than pRNFL, suggesting it as a superior biomarker to monitor glaucoma at this stage. For damage mechanisms, OCT-A might uncover the vascular dysfunction role in glaucoma. In a recent study, Chen et al. [21] demonstrated OCT-A detectable vascular alterations prior to structural and functional loss in the normal hemifield of the eyes with glaucoma. This might contribute to the investigation of the hypothesis that vascular dysfunction might precede other structural and functional changes and hence help to determine an ideal window to implement therapeutic agents to salvage dysfunctional RGC. In summary, neither method, OCT nor OCT-A, can substitute the other and their combined usage in glaucoma promises an optimization early diagnosis, progression monitoring, and the elucidation of pathomechanisms. This approach might be extended further by longitudinally designed studies and the improvement of OCT-A technology in terms of accuracy and analysis. In addition, we have the option to integrate functional measures from non-invasive electrophysiology with these imaging approaches, as detailed below.

## Non-invasive electrophysiology

We will review methods offered in clinical visual electrophysiology and their relation to glaucoma management, drawing heavily on previous reviews [2, 22, 23] and especially on [24]. The latter publication describes all electrophysiological methods in detail and was co-authored by one of us. This section will conclude with an example of an experimental approach to utilize non-invasive electrophysiology to uncover potential damage mechanisms, here the relation of RGC function and IOP changes.

Clinical visual electrophysiology comprises a large set of methods with an associated alphabet soup of acronyms. Table 1 gives an overview of these with our estimation to what degree these are a useful biomarker in glaucoma, be it (early) diagnosis or monitor for progress or treatment efficacy. Our estimate of usefulness is based on the generator (e.g., the photoreceptors are not or very little affected), on the published results, and in part on our own experience.

**Table 1:** Clinical visual electrophysiology methods with estimated usefulness in glaucoma diagnosis and/or monitoring.

Short	Full name	Stimulus	Reflecting function of	Glaucoma relevant
Full-field flash ERG	Electroretinogram	Flash	a-wave: rods; b-wave: bipolar cells	No
		Flash, flicker	Cones	No
mfERG	Multifocal ERG	Local flashes	Cones and bipolar, spatially resolved	No
STR	Scotopic threshold response	Flash	Retinal ganglion cells	No
EOG	Electrooculogram	Light rise	Retinal pigment epithelium (driven by metabolic demands from receptors)	No
PhNR	Photopic negative response	Flash	Retinal ganglion cells	Yes
PERG	Pattern ERG	Pattern change	Retinal ganglion cells	Yes
mfPERG	Multifocal PERG	Local pattern change	Retinal ganglion cells, spatially resolved	Yes
VEP	Visual evoked potential	(Flash) or pattern	V1 (and higher areas)	(Yes)
mfVEP	Multifocal VEP	Local pattern change	V1, spatially resolved	Yes

In accordance with Table 1, we will only report here on the photopic negative response (PhNR) component of the ERG, the pattern ERG, and the visual evoked potential (VEP). All three come in “multifocal” variants. Multifocal stimulation [25] is a tool that, beginning about 25 years ago, advanced the field enormously by allowing spatially resolved functional imaging without undue increase in recording time. Basically, multifocal stimulation presents a large set of orthogonal stimuli, thus stimulating about half of all locations quasi-simultaneously, and extracting the local response via crosscorrelation of the global response with the local stimulation information. The “classic” full-field electroretinogram and its multifocal variant have been applied with many a variation in glaucoma, but ultimately with relatively little success [24], so will not be covered here.

## PhNR

**Fig. 1:**
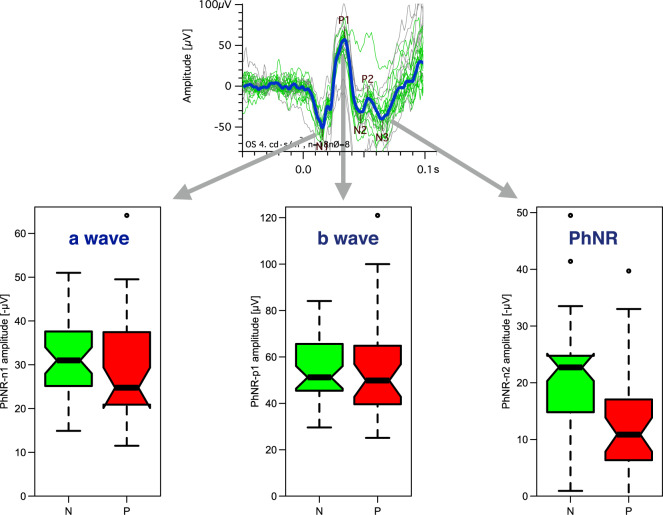
Structures in the flash ERG (top) and their affection in glaucoma (bottom); based on data from [101]. The top trace example shows in green every single accepted trace, in gray the rejected ones (not meeting a time/amplitude window criterion), and in blue the average. Below are group boxplots. Green (left): controls, red: glaucoma eyes. The a- and b-waves (the first two main structures) are not significantly changed by glaucoma, but the PhNR is markedly reduced (color figure online).

The PhNR [26, 27] is recorded with corneal (or skin) electrodes usually in response to a (red) flash on a blue background. The adapting background and flash color are chosen to select cone over rod responses. In the resulting flash response the PhNR is a shallow negative excursion after the b-wave, peaking around 70 ms (Fig. 1); its amplitude, and its normalized amplitude to the b-wave (PhNR ratio), is reduced in glaucoma [28, 29]. Recent reports combined the conventional PhNR paradigm with multifocal stimulation (mfPhNR) to assess the topography of the retinal response. They applied a variety of multifocal stimulation modes ranging from rapid [30, 31] to slow stimulation [32, 33]. In a direct comparison of these different modes, rapid multifocal stimulation appeared beneficial for the early detection of glaucoma with the mfPhNR [34].

### Disadvantages of the PhNR

- Mildly invasive, electrodes at the cornea or near the eye.
- With current protocol, pupil dilation is required.
- Patient preparation ½ h, recording time ≈5 min

### Advantages of the PhNR

- No high-quality optical imaging required (no trial glasses, accommodation irrelevant).
- Direct indicator of ganglion cell function.
- Along glaucoma progression, PhNR amplitude may floor later than pattern electroretinogram (PERG) (see section “Floor effects and dynamic range” below).
- Recent progress with rapid stimulation suggests improved sensitivity.

## PERG

The PERG [35–37] is recorded with corneal (or skin) electrodes in response to pattern change on a VDU. For transient (slow) stimulation, its major components are positivity at ≈50 ms (P50) and negativity (N95) at ≈100 ms after pattern change. Both components arise probably from the RGCs, with the P50 likely representing input activity to the RGCs (EPSPs and IPSPs) and the N95 representing spiking activity. PERG amplitude is reduced in glaucoma, especially with rapid stimulus presentation [38]. Interestingly, with advancing glaucoma its peak time decreases [39, 40], which has been overlooked due to erroneous interpretation of phase in steady-state responses [40].

### “Local” origin of the PERG—a tool to disentangle RGC axon defects from retinal ones

**Fig. 2:**
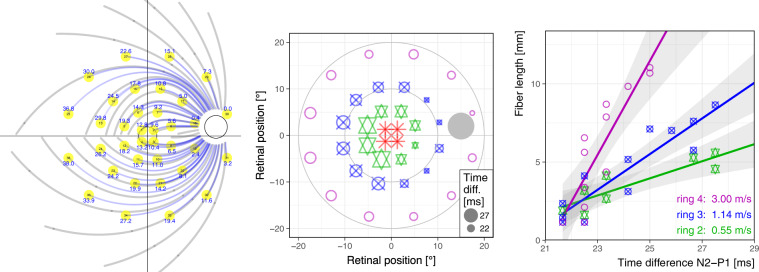
Local origins of PERG components. Left: locations of mfPERG stimuli (yellow) in relation to fiber tracks. Center: time difference (∆*t*) between local P50 and N95 mfPERG peaks reflected in symbol size; based on data from [43]. Right: two factors explain 82% of ∆*t*’s variance: eccentricity (color/shape) and length of fiber track from stimulus locus to the optic disc (color figure online).

It is well established that the PERG (all of its components) originates from the RGCs [41, 42]. The RGCs are large, however, with their axons terminating in the lateral geniculate nucleus. Using the multifocal PERG, Bach et al. [43] found timing differences of the P50 versus the N95 which depended on retinal location. The findings point to the sharp bend of the axons at the optic disk as the locus for the electric sources of the N95. This opens up the possibility of using different components of the PERG to assess local function across the retina (P50-related) as opposed to the optic disk (N95-related) Fig. 2.

### Disadvantages of the PERG

- Mildly invasive, electrodes at the cornea or near the eye.
- Adequate optical imaging necessary (trial glasses, accommodation).
- Patient preparation ½ h, recording time ≈5 min.
- Sensitive early, then saturated; thus not useful as a marker in later glaucoma stages.
- For stimulation, no LCD displays can be used.

### Advantages of the PERG

- A direct indicator of ganglion cell function.
- Affected very early (probably before marked field changes are present).
- Large literature body available including longitudinal studies that find success in early diagnosis.
- More sensitive with rapid pattern changes (>10 times per second), allowing Fourier-based automatic. analysis without the need for individual peak assessment.

## VEP

The VEP, being a long-standing well-developed tool with little invasiveness, has been assessed many times for its use in glaucoma. It is a given that glaucoma will affect the VEP: Glaucoma leads to ganglion cell loss, and the VEP, representing visually-driven cortical activity, will necessarily be affected. The crucial aspect is its sensitivity and specificity. The VEP signal can be delayed and/or reduced in amplitude. For instance, a very positive report [44] reported that “the P100 (peak) times were delayed in … 85% ocular hypertension eyes and … 100% OAG eyes”. One of us wryly commented that may have been an “auspicious patient group” [45] and indeed [46] did not report similar findings. VEP amplitude reductions may be more relevant, especially in the mfVEP (see below).

### Special techniques can increase the value of the VEP

- The blue-sensitive pathway is thought to be especially vulnerable in glaucoma, probably due to its relatively low dynamic range [47]. Targeting specifically this pathway, a sizable longitudinal study [48] found VEP peak time changes 2 years before morphologic changes became evident. This has not led to widespread applications, possibly due to the difficulty to isolate this pathway in spite of yellowing lenses with age.
- “ic-VEPs” (isolated-check VEPs, [49]) sweep contrast (or at least use 10% and 80%) and use the signal-to-noise ratio as a marker. With a commercial version (using an OLED monitor, so laudably without luminance artifacts), in a 140-eyes cross-sectional study, Kolomeyer et al. [50] reported AUCs of up to 0.79, depending on whether favoring sensitivity or specificity. They also found progressive VEP affection from pre-perimetric over early and moderate to severe glaucoma.
- The “SD-tVEP” (short duration transient VEP, [51]): a typical 2-eye-recording time of ≈7 min is reduced to 1½ min. This impressive achievement (by fitting the response to the model of a typical VEP trace, derived from the ERG’s oscillatory potential) is practical of some use, but then realistic total setup times add a large overhead. Using this tool, Prata et al. [52] examined 25 patients with asymmetric glaucoma. They report significant findings and correlations for VEP latency and (a little less) for VEP amplitude to morphological parameters. Since the authors performed over 20 *t*-tests and further 16 correlation tests without correction for multiple testing, these findings await rigorous re-testing.

### Disadvantages of the VEP

- High interindividual amplitude variability.
- Very few longitudinal studies are available.
- LCD displays should not be used for stimulation (but are).

### Advantages of the VEP

- Little invasiveness (only scalp electrodes).
- Useful for monitoring (no saturation like PERG).

## mfVEP

In principle, the multifocal VEP (mfVEP) could serve as perfect objective perimetry. Small obstacles are the necessity of good retinal imaging with respect to refraction and accommodation and steady correct fixation. A major problem false-positive scotomata. Due to the complicated folding of V1, for some field locations, the corresponding activity in V1 does not sufficiently project to the scalp electrodes, even if multiple channels [53] are used. A detailed discussion is available here [24], and the conclusion by Graham [54] still holds “The mfVEP is particularly useful for investigating patients with field loss that does not match the clinical picture, either because they are poor performers on subjective tests or there is a suspicion of other pathology.” as exemplified in [55]. In conclusion, mfVEPs do not out-perform subjective perimetric tests in the detection of VF defect and consequently do not provide means for early detection of glaucoma. However, mfVEPs are of assistance in resolving unexplained or inconclusive VF defects detected in subjective tests. Current developments might make this application field available for broader use, e.g., by providing portable compact brain-computer interfaces with head-mounted displays and integrated eye movement monitoring for mfVEP-based VF assessment [56].

### Disadvantages of the mfVEP

- False-positive scotomata due to the convoluted cortical folding and the ensuing loss of dipole projection on surface electrodes.

### Advantages of the mfVEP

- Objective perimetry.

### A provocative test model using PERG and positional change

Electroretinographic measures of RGC function might help to disentangle the interplay of the RGC function and risk factors for glaucoma, specifically the intraocular pressure (IOP), not only for elucidating damage mechanisms but also for defining the onset of glaucoma. An attractive maneuver to decipher the IOP-RGC relationship is to manipulate IOP by employing body-position-induced physiological changes and to manifest its impact on PERG, i.e., as an indicator of RGC function.

Ventura et al. [57] probed the susceptibility of RGC during −10° head down body tilt (HDT) in GS and early glaucomatous patients and demonstrated worsening of the PERG signal in a subset of this patient. It was concluded that HDT might serve as a physiological stressor on dysfunctional RGC, i.e., reduced PERG [57]. The high susceptibility of RGC induced by this physiological stressor might serve as a time window to implement therapeutic agents to prevent permanent structural loss [58]. This research group extended their study by following up GS and revealed that baseline hemodynamic and PERG changes obtained 5 years earlier predicted the conversion into glaucoma, i.e., pRNFL thickness loss in OCT.

**Fig. 3:**
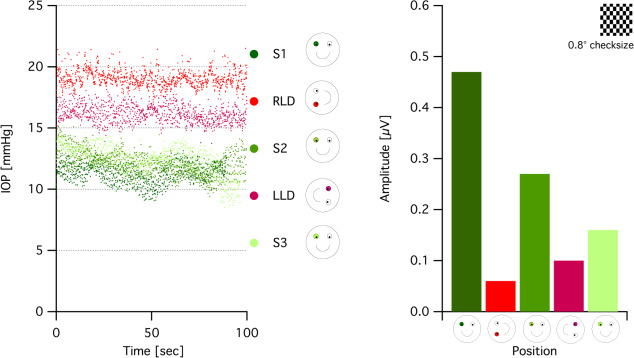
Continuous IOP and PERG measurements during postural changes. Left: continuous IOP measurements of a representative glaucoma patient with a monocular eyemate-IO® sensor implant (right eye), for different body positions. Right: corresponding PERG amplitudes to 0.8° checksize during each position in the same participant. The patient was positioned in the following order: S1 initial setting, RLD right lateral decubitus, S2 setting 2, LLD left lateral decubitus, S3 sitting 3. Data from [59] (color figure online).

Another recent study [59] utilized the posture-induced IOP manipulation with a simpler positioning approach, i.e., lateral decubitus posture (LDP) Fig. 3. The authors coupled PERG recordings with a simultaneous tracking of IOP exploiting a novel IOP sensor (eyemate-IO®, Implandata Ophthalmic Products GmbH, Hannover, Germany) co-implanted in glaucoma patients during cataract surgery; the eyemate-IO sensor proved to be safe, well-tolerable, and functional [60]. Continuous IOP measurement allows for the investigation of short-term fluctuations of IOP and its direct effect on RGC function during simultaneous PERG recordings: the robustness of steady-state PERG allowed simultaneous recording with the continuous IOP measurement due to its frequency-based amplitude extraction at 15 Hz that is dissociated from the sensor frequency intrusions at 9 Hz [59]. The LDP induced a significantly higher IOP in the lower eyes in accordance with other studies [61, 62], in both glaucoma patients with the eyemate-IO sensor and healthy controls. The increased IOP was accompanied by reversible reductions of RGC responses determined via PERG both in controls and glaucomatous eyes. This opens doors to scrutinize the relationship of IOP and RGC function with a novel approach employing state-of-art methods of IOP and PERG measurements. This model could be further utilized as a provocative test in GS and to decipher mechanisms in glaucomatous damage such as asymmetrical VF defects [57].

## Commonalities and complementarities of the modalities OCT and electrophysiology

As evident from the above, both OCT and electrophysiology bear great potential for improved management of glaucoma. Assessing overlap and complementarities of both approaches will help to identify rewarding targets of combined applications of both techniques with the aim to establish reliable endpoint biomarkers along the disease continuum, particularly for glaucoma conversion and advanced glaucoma. This might offer a better understanding and quantification of glaucomatous damage with respect to the functional and structural relationship. This will in turn optimize glaucoma detection by tailoring tests according to the stage of the disease. In order to identify these targets, we firstly evaluate relevant application fields for commonalities and subsequently review recent studies of combined OCT and electrophysiological research in glaucoma.

### Longitudinal studies: endpoint biomarkers for glaucoma conversion

At the earliest stages of glaucoma, functional damage might not be detectable on SAP, albeit being the most commonly used to monitor glaucoma progression, due to its subjective nature and variability of results [63]. Therefore, the combined use of alternative functional tests, i.e., ERG, and structural tests, i.e., OCT, might lead to timely identification of these cases and improve glaucomatous management. For a comprehensive approach and optimal design, longitudinal studies following patients with questionable glaucomatous stage, i.e., glaucoma suspect (GS), are critical to examine structural and functional damage and might provide evidence about the utility of these measures as sensitive endpoints for glaucoma conversion.

#### Functional biomarkers

The PERG is a well-established and sensitive biomarker of RGC function [41, 64] employed for the detection of early glaucoma [34, 65–69]. Longitudinal studies following up early glaucoma cases, i.e., GS and ocular hypertension patients (OHT) substantiated the evidence of the PERG utility in glaucoma diagnosis. Bode et al. [67] followed OHT prospectively for ≈10 years and found that PERG unveiled glaucoma conversion 4 years earlier before the appearance of any detectable VF changes. Banitt and colleagues [70], on the other hand, anticipated permanent structural loss of RGC in GS several years earlier by demonstrating reversible RGC dysfunction on PERG. A recent study by Gordon et al. [69] extended and supported these findings by demonstrating mutual functional, PERG, and structural, pRNFL, changes in a subset of GS over 10 years. Lack of correlation between pRNFL and PERG changes over years in this longitudinal retrospective study [69] indicated that both measures independently reflect different damage mechanisms in glaucoma.

#### Structural biomarkers

OCT has been widely utilized to diagnose glaucoma [71, 72] and found to be useful to assess the risk of glaucoma conversion in GS. Zhang et al. [10] demonstrated that 70% of glaucoma converters had abnormal or borderline GCC or pRNFL variables at baseline during a 41 ± 23 months of follow-up. Another study [73] with a median follow-up of 6.3 years reported 19% of GS eyes had abnormal pRNFL 8 years before the visual loss. Medeiros et al. [74] also followed GS for a median of 6.7 years and concluded that RGC loss in glaucomatous eyes compared to controls had 28% loss on average at the earliest VF defect on SAP. With the inclusion of a disease continuum from GS to advanced glaucoma, other recent longitudinal studies showed that OCT endpoints, i.e., pRNFL [75, 76] and GCC [75], had good detection of progression in early glaucoma, whereas macular measures, i.e., GCC, were better biomarkers in advanced glaucoma stages.

We conclude that functional and structural assessment through ERG measures and OCT technologies provide complementary information that might, in combination, optimize the early detection of glaucoma.

### Floor effects and dynamic range: endpoint biomarkers for advanced glaucoma

#### Functional biomarkers

Following progression in advanced glaucoma is quite challenging due to the variability of VF defects. To reduce test-retest VF variability in advanced damage, some [77, 78] recently suggested limiting the sensitivities of VF testing to at least 19 dB. Another study demonstrated that VF variability is not necessarily related to low sensitivities in fundus-tracked perimetry [79]. Moreover, one study [80] demonstrated a decrease in VF progression when the glaucomatous damage increases, i.e., reduction of the remaining measurable visual function, a floor effect [80]. ERG measures of visual function do not offer superiority for monitoring later stages of glaucoma progression, where PERG decreases early and its amplitude might not decline much further with disease progression [22, 40, 70]. The multifocal PhNR may floor later than PERG as suggested by progressively reduced mfPhNR amplitude in more advanced glaucomatous stages and its reduction correlated with the disease severity [30]. However, Machida et al. [81] reported that focal PhNR is not suitable to follow advanced glaucomatous changes. These unresolved findings highlight the unmet need for better methods to detect glaucoma progression rather than the trend analysis of VF testing.

#### Structural biomarkers

There is a growing body of evidence that pRNFL thinning is not observable anymore in advanced glaucomatous cases, again a floor effect [82, 83]. It is, however, of paramount importance to follow the treatment and the stability of glaucoma in these patients, particularly as they are most liable to eventual blindness. Floor avoidance has recently gained growing attention due to advances in imaging technology of the structural indexes in glaucoma. Bowd and colleagues [84] have reported that the macula and its surrogate biomarker, i.e., GCIPL, is spared from the floor effect and might be a better measure for following advanced glaucomatous eyes. Miraftabi et al. [85] reported that the dynamic range of OCT parameters of the macula did not exceed 8–10 dB of total deviation loss. In fact, all OCT structural measures have floor effects after an 8–10 dB decline in perimetric sensitivity at the corresponding test locations. Further, central RGC damage might manifest as early as pRNFL damage, but the abundance of RGC in the macula is the reason, why the macula is the only retinal region to follow glaucoma at advanced stages without significant variability with disease worsening [2, 86]. In more advanced cases, macular thickness measures might also reach the measurement floor and OCT loses its utility for detection of disease deterioration [2]. In a recent study, Moghimi et al. [87] assessed the measurement floors and dynamic ranges of OCT and OCT-A measures and demonstrated that VD of the macula is a promising tool in progression detection, since it showed no detectable floor measurement in comparison to earlier floor measurements of macular and peripapillary thickness; however, the latter thickness measures have more steps. A number of steps, i.e., the no. of steps between normal thickness and thickness floor, is another concept complementary to dynamic range and used for objective monitoring and staging of the disease.

In a recent review of longitudinal studies in glaucoma progression [88], it was concluded that structural and functional measures change concordantly. We, therefore, believe that functional and structural measures besides the new vascular indexes should be utilized jointly for the assessment of the progression along the disease continuum.

## Benefits of multimodal assessments

Electrophysiological approaches in glaucoma research and diagnostics benefit from the combination with other techniques. Given the considerable variability of structural and functional tests [89], multimodal assessments might offer a chance to improve glaucoma management and to understand the underlying pathophysiology. Below, we explore the scope of using ERG measures in optimization of glaucoma detection as well as the advantages when combined with state-of-the-art advances in ophthalmology i.e., OCT-A.

### Damage mechanisms: macular damage in glaucoma

Traditionally, glaucoma was thought to affect the peripheral VF; however, Bach et al. [65, 90] investigated glaucomatous damage utilizing VF mean deviation (MD) and PERG and proposed that glaucoma starts with PERG-detectable panretinal damage but progresses preferentially with arcuate defects shown both on VF and PERG. They reported that large VF defects showed abnormal PERG, but eyes with normal central VF simultaneously with peripheral VF defects demonstrated abnormal PERG, too. The authors concluded that PERG could detect central macular damage albeit in the absence of central VF defects on conventional automated perimeters. Electrophysiologically this was substantiated with the multifocal PERG [91, 92]. A recent study of mfPhNR [34] in glaucoma diagnostics found no central VF damage in glaucoma, in contrast to findings by others [30].

Years later, a substantial body of literature established early involvement of and even initial defects in the macula in glaucoma [7, 93]. Hood et al. [7] furthered the understanding of early macular involvement in glaucomatous damage employing structural tests of the OCT. As reviewed by Hood [7], it was affirmed that macular damage is common in early glaucoma that might be missed in VF 24–2 tests. This conclusion was grounded on many reasons including, (i) inferior GCIPL being more susceptible to glaucomatous damage and (ii) the central 6° grid of 24–2 VF missing this region. In fact, the first PERG report [65] on early involvement of central retinal in glaucoma was 15 years prior to the structural evidence, this confirms the PERG’s potential to the elucidation of pathomechanisms in glaucoma.

### ERG and OCT angiography: structure-vascular and functional relationship

As a consequence of the prominent developments in OCT technology, OCT might be of considerable help for the characterization not only of structural retinal changes in glaucoma [94] but also of vascular changes [95]. Finally, electroretinographic approaches provide highly sensitive readouts of glaucoma-related damage. This prompts the question of the complementarities of these different approaches and the potential benefit of their combined use in order to provide insights and hallmarks about the damage mechanisms in glaucoma. An example of the benefit of such a multimodal assessment of glaucoma is given below on research that addresses the two popular theories on glaucoma pathogenesis, i.e., the “mechanical” vs the “vascular” theory.

RGC damage in glaucoma might be attributed to elevated IOP, exerting mechanical stress on the optic nerve head, i.e., “mechanical theory” [96, 97]. However, a subset of glaucoma patients deteriorates in spite of good IOP control proposing other damage mechanisms; among those is the loss of RGC due to vascular dysfunction, i.e., the “vascular theory” [98]. Although IOP is the main risk factor, the interplay of these two mechanisms [99] might be implicated in the primary open angle glaucoma (POAG), the most common type of glaucoma. In normal-tension glaucoma (NTG), on the other hand, the vascular dysfunction in its pathogenesis dominates [100].

To elucidate these pathomechanisms, ERG measures of vision function might play a pivotal role. In a study combining PhNR of ERG with OCT-A in NTG [18], macular measures of vascular alteration, vessel density (VD), in early-stage NTG patients were only associated with the functional measure of RGC, i.e., PhNR amplitude, and not with structural OCT measures. This might be due to preferential early vascular damage to RGCs preceding structural damage in NTG and subsequent influence of RGC function. Another study extended this finding and corroborated the use of ERG measures, i.e., mfPhNR, to decipher pathomechanisms in POAG [19]. Here, the mfPhNR as an index of RGC function in POAG showed a higher association with structural parameters, i.e., macular GCIPL and pRNFL, than vascular macular and peripapillary VD [19]. This might point to structural damage preceding vascular damage in POAG—in contrast to NTG. In conclusion, this hints at possibly stronger importance of vascular mechanisms in NTG and of mechanical mechanisms in POAG, underlining the potential of the combined multimodal assessment of retinal damage in glaucoma employing OCT, OCT-A, and electroretinography. Future studies, ideally longitudinal ones, have the potential to assess whether such multimodal assessments are also of benefit for the early detection of glaucoma.

## Summary and Conclusion

Electrophysiology functional measures of vision provide an integral pivotal role in glaucoma management along with state-of-the-art ophthalmology technologies, i.e., optical coherence tomography/angiography as evidenced by the detection of early glaucoma and elucidations of damage mechanisms. Future directions employing longitudinally designed studies might corroborate the utility of multimodal approaches in the current standards of glaucomatous care for the sake of early implementation of therapy and prevention of blindness. The translation of multimodal approaches into practice might potentially add a major diagnostic dimension to the currently established tests in glaucoma as well as provide insights to several unanswered inquiries about glaucoma pathogenesis.
